# Crystal structures of tri­chlorido­(4-methyl­piperidine)gold(III) and two polymorphs of tri­bromido(4-methyl­piperidine)­gold(III)

**DOI:** 10.1107/S2056989024002822

**Published:** 2024-04-18

**Authors:** Cindy Döring, Peter G. Jones

**Affiliations:** a Institut für Anorganische und Analytische Chemie, Technische Universität Braunschweig, Hagenring 30, D-38106 Braunschweig, Germany; University of Kentucky, USA

**Keywords:** crystal structure, gold, methyl­piperidine, hydrogen bonds, polymorph

## Abstract

Tri­chlorido­(4-methyl­piperidine)­gold(III), [AuCl_3_(C_6_H_13_N)], **1**, crystallizes in *Pbca* with *Z* = 8. Tri­bromido­(4-methyl­piperidine)­gold(III), [AuBr_3_(C_6_H_13_N)], **2**, crystallizes as two polymorphs, **2a** in *Pnma* with *Z* = 4 (imposed mirror symmetry) and **2b**, which is isotypic to **1**. The Au—N bonds *trans* to Cl are somewhat shorter than those *trans* to Br, and the Au—Cl bonds *trans* to N are longer than those *cis* to N, whereas the Au—Br bonds *trans* to N are slightly shorter than the *cis* bonds. The methyl and Au*X*_3_ groups (*X* = halogen) occupy equatorial positions at the six-membered ring. The packing of all three structures involves chains of mol­ecules with offset stacking of the Au*X*_3_ moieties associated with short Au⋯*X* contacts; for **1** and **2b** these are reinforced by N—H⋯*X* hydrogen bonds, whereas for **2a** there are no classical hydrogen bonds and the chains are inter­connected by Br⋯Br contacts.

## Abstract 2

All three structures involve chains of mol­ecules linked by Au⋯halogen contacts; these are accompanied by hydrogen bonds or halogen⋯halogen contacts.

## 1. Chemical context

We have published a series of articles describing the structures of amine complexes of gold. The three most recent, Parts 12–14 in the series, concerned gold(I) and gold(III) derivatives of piperidine and pyrrolidine (Döring & Jones, 2023*a* ▸), gold(I) complexes of morpholine (Döring & Jones, 2023*b* ▸) and gold(I) complexes of methyl­piperidine (Döring & Jones, 2024 ▸). An extensive introduction, with details of previous results, may be found in Part 12 and will not be repeated here. Here we present the structures of the two 4-methyl­piperidine complexes of gold(III) trihalides, namely tri­chlorido­(4-methyl­piperidine)­gold(III) **1** and tri­bromido­(4-methyl­piperidine)­gold(III) **2**. The ligands piperidine and 4-methyl­piperidine are henceforth abbreviated to ‘pip’ and ‘4-Me-pip’. 
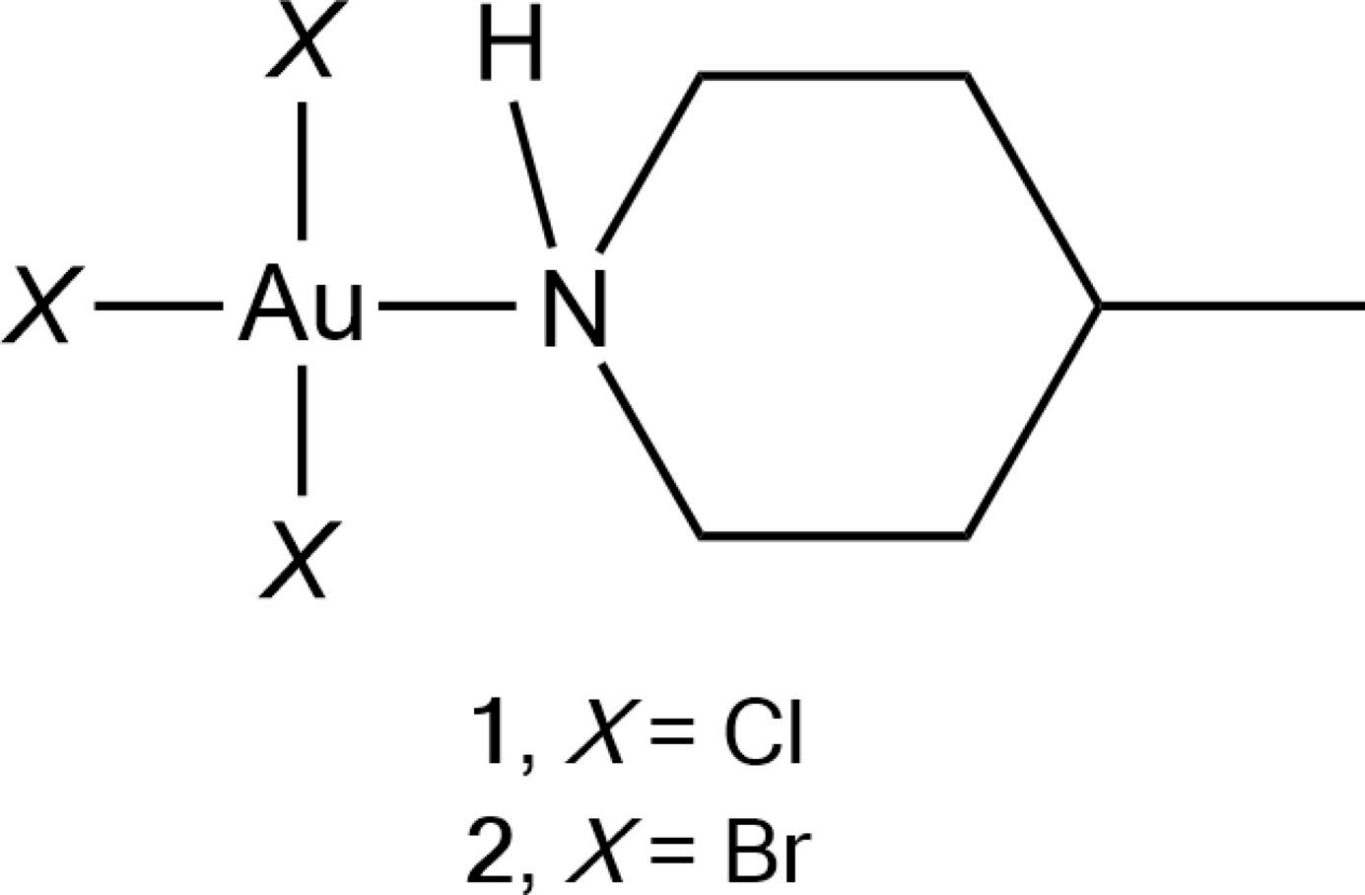


## 2. Structural commentary

**Figure 1:**
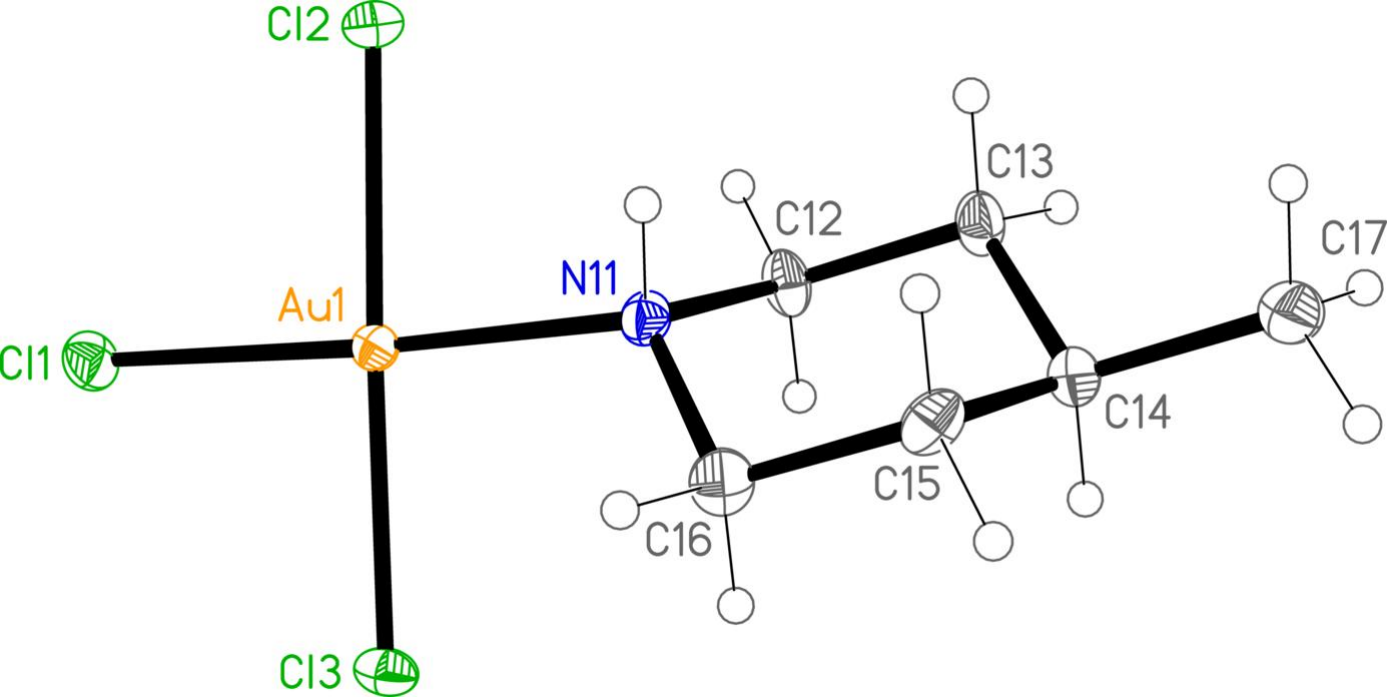
The structure of compound **1** in the crystal. Ellipsoids correspond to 50% probability levels.

The mol­ecular structures of **1**, **2a** and **2b** are shown in Figs. 1 ▸–3 ▸ ▸. Compound **2** crystallized as two polymorphs in the space groups *Pnma* (**2a**) and *Pbca* (**2b**); the former displays crystallographic mirror symmetry, whereby the mirror plane contains the gold and bromine atoms, the NH group, the carbon at C-4 and the methyl carbon (these atoms are numbered for **2a** as C14 and C15). For all three structures, the halogen atoms are numbered such that *X*1 (*X* = halogen) is *trans* to the ligand nitro­gen atom N11. Structures **1** and **2b** are isotypic. The geometry at the gold atoms is as expected square planar. Bond lengths and angles (Tables 1 ▸–3 ▸ ▸) may be considered normal. The Au—N bonds *trans* to Cl are somewhat shorter than those *trans* to Br, and the Au—Cl bonds *trans* to N are longer than those *cis* to N, whereas the Au—Br bonds *trans* to N are slightly shorter than the *cis* bonds. Similar trends were observed for (pip)AuCl_3_ and (pip)AuBr_3_ (Döring & Jones, 2023*a* ▸).

The relative orientation of the ligand and the Au*X*_3_ unit is described by the torsion angles *Xn*—Au1—N11—H01 and *Xn*—Au1—N11—C, where *n* = 2 or 3 (torsion angles for *n* = 1 are meaningless because the sequence *X*1—Au1—N1 is linear). We observe two distinct types: either one angle *Xn*—Au1—N11—H01 is approximately zero, corresponding to a short H01⋯*Xn* contact that might be considered an intra­molecular hydrogen bond, and the smallest absolute *Xn*—Au1—N11—C angle is around 60°, or the angle *Xn*—Au1—N11—H01 is approximately 30–40° and the smallest absolute *Xn*—Au1—N11—C angle is around 30°. The former type applies to (pip)AuCl_3_ and **2a** [where Br2—Au1—N11—H01 is exactly zero by symmetry and H01⋯Br2 is 2.71 (6) Å], and the latter to (pip)AuBr_3_, **1** and **2b**.

As would be expected for bulky substituents attached to cyclo­hexane-type rings, the methyl groups and the Au*X*_3_ moieties occupy equatorial positions, with torsion angles C—C—N—Au and C—C—C—C_meth­yl_ around ±180°. Our previous two papers however include several structures where a gold(I) atom occupies an axial position in similar mol­ecules. The ‘normal’ equatorial positions observed for **1**, **2a** and **2b** may be associated with steric effects, which should be greater for the larger Au*X*_3_ moieties compared to the linear gold(I) centres.

## 3. Supra­molecular features

**Figure 4:**
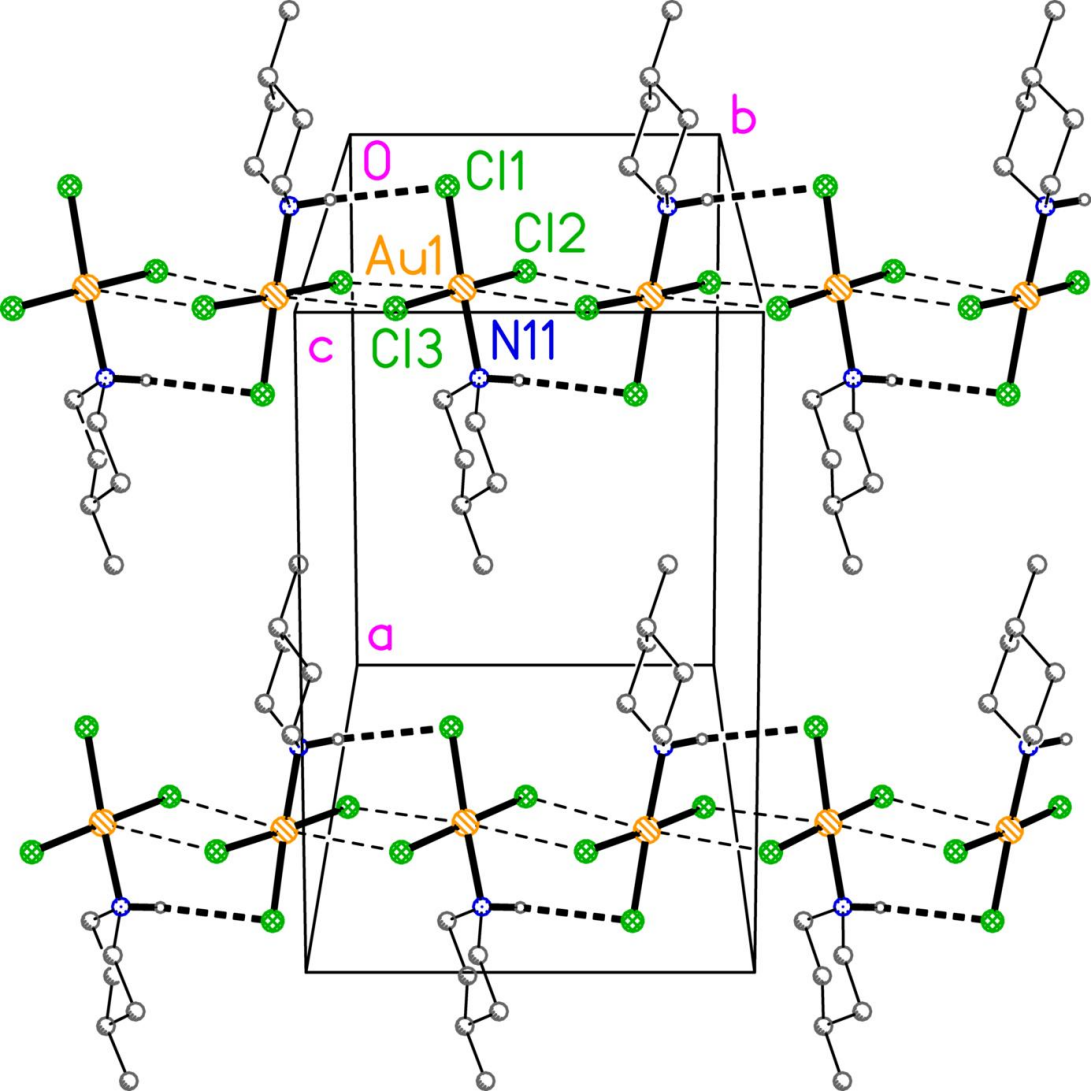
Packing diagram of compound **1** viewed approximately parallel to the *c* axis (but rotated by *ca* 15° around the horizontal axis for clarity) in the region *z* ≃ 0.125, showing two chains of mol­ecules parallel to the *b* axis. Dashed lines indicate H⋯Cl hydrogen bonds (thick) or Au⋯Cl contacts (thin). Hydrogen atoms not involved in hydrogen bonding are omitted. Atom labels indicate the asymmetric unit. Similar chains are formed in the regions *z* ≃ 0.375, 0.625 and 0.875.

For compound **1**, the main inter­molecular contacts are the hydrogen bond N1—H01⋯Cl1( 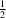 − *x*, 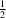 + *y*, *z*, the *b* glide operator) and the two Au⋯Cl contacts Au1⋯Cl3 (same operator) = 3.2980 (10) Å and Au1⋯Cl2( 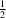 − *x*, − 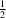 + *y*, *z*) = 3.3604 (10) Å that correspond to an offset stacking of the AuCl_3_ moieties. These combine to form chains of mol­ecules parallel to the *b* axis (Fig. 4 ▸). In the isotypic **2b**, the corresponding Au⋯Br distances are 3.4060 (5) and 3.5018 (5) Å.

**Figure 5:**
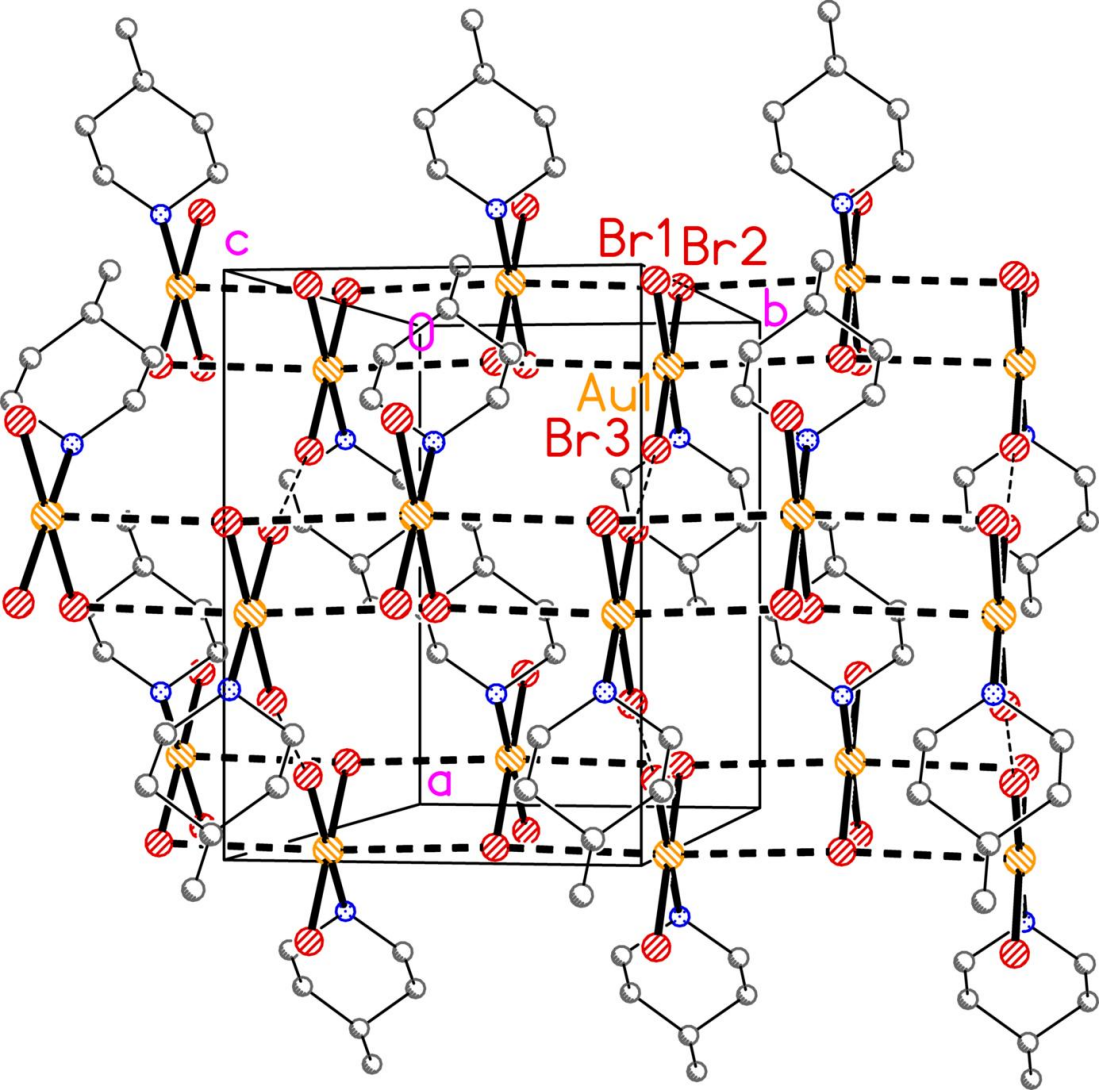
Packing diagram of compound **2a** viewed aproximately parallel to the *c* axis (but rotated by *ca* 10° about the vertical axis for clarity), showing three chains of mol­ecules parallel to the *b* axis. The chains are centred on the regions (*x*, *z*) = (0, 0), (1, 0) and (1/2, 1/2). Dashed lines indicated Au⋯Br contacts (thick) or Br⋯Br contacts (thin); the latter are shown more clearly in Fig. 6 ▸. Atom labels indicate the asymmetric unit.

**Figure 6:**
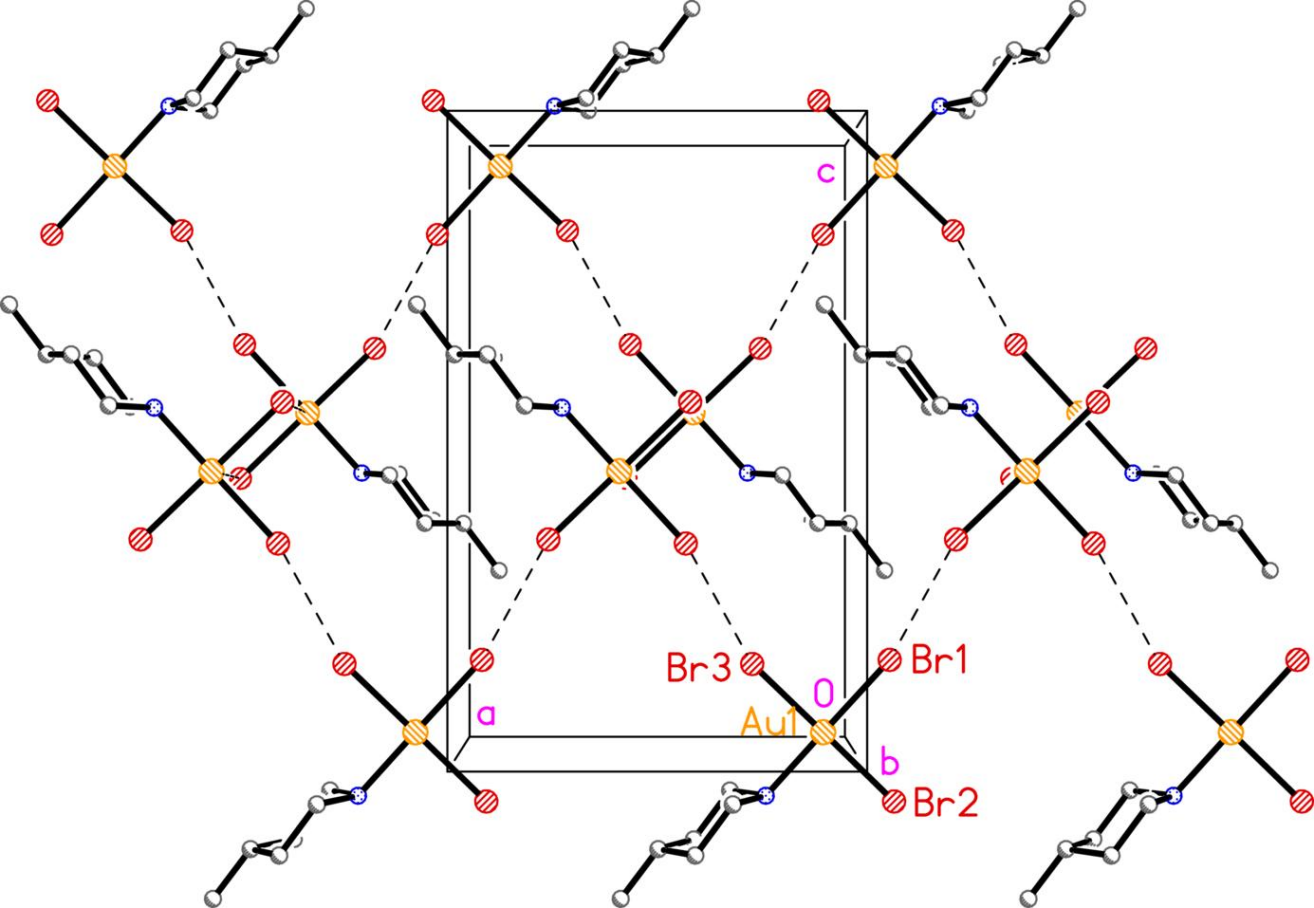
Packing diagram of compound **2a** showing two zigzag chains of mol­ecules parallel to the *b* axis; the lower chain is centred in the mirror plane at *y* = 0.75 and the upper chain in the plane at *y* = 0.25. Dashed lines indicated Br⋯Br contacts (or, just visible, Au⋯Br contacts linking the two chains in the direction into the paper). Atom labels indicate the asymmetric unit.

Compound **2a** forms chains analogous to those of **1**, with Au1⋯Br2(−*x*, 1 − *y*, −*z* and −*x*, 2 − *y*, −*z*) = 3.5847 (2) Å; these run parallel to the *b* axis (Fig. 5 ▸). The chains are crosslinked by short Br⋯Br contacts involving one *cis* (to N) and the *trans* Br atom, with Br1⋯Br3(− 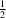 + *x*, *y*, 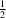 − *z*, the *a* glide operator) = 3.3686 (6) Å and angles Au1—Br1⋯Br3′ = 166.26 (3) and Au1—Br3⋯Br1′ = 162.77 (3)°. These contacts are indicated in Fig. 5 ▸ but are shown more clearly in Fig. 6 ▸; they link the mol­ecules to form chains parallel to the *b* axis. The NH group is not involved in inter­molecular hydrogen bonding.

All three structures also display C—H⋯ *X* contacts that might be inter­preted as ‘weak’ hydrogen bonds (Tables 4 ▸–6 ▸ ▸), but none of these is strikingly short. These (and other) weak inter­actions might well contribute significantly to the packing energy, but it is difficult to incorporate them in easily inter­pretable packing diagrams.

## 4. Database survey

The searches employed the routine ConQuest (Bruno *et al.*, 2002 ▸), part of Version 2023.3.0 of the Cambridge Database (Groom *et al.*, 2016 ▸). A search for short Cl⋯Cl contacts between mol­ecules *L*AuCl_3_ (*L* = any atom) gave 51 hits (59 independent mol­ecules) with contact distances from 3.086 to 3.37 Å and an average Au—Cl⋯Cl angle of 152.9°. A similar search for *L*AuBr_3_ (*L* = any atom) gave 28 hits (36 independent mol­ecules) with contact distances from 3.26 to 3.67 Å and an average Au—Br⋯Br angle of 150.7°. The upper bounds for the contact distances in both cases correspond to the double van der Waals radii as stored in the CCDC. For both sets of results, the *cis* (to *L*) halogen atoms were more often involved than the *trans* halogen atoms (the latter corresponding to *X*1 in the structures presented here); for *X* = Cl there were 9 contacts of the form *trans*/*trans*, 5 *cis*/*trans* and 37 *cis*/*cis*, and the corresponding values for *X* = Br were 4, 7 and 25. In many cases, the Au—*X*⋯*X* angles were equal by symmetry, and both values were used to calculate the average values.

## 5. Synthesis and crystallization

The starting materials of choice would be the gold(I) complexes (4-Me-pip)Au*X*, but these exist in the ionic form [(4-Me-pip)_2_Au][Au*X*_2_] rather than as neutral mol­ecules (Döring & Jones, 2024 ▸).

*Tri­chlorido­(4-methyl­piperidine)­gold(III)* (**1**)

A solution of bis­(4-methyl­piperidine)­gold(I) di­chlorido­aurate(I) (310 mg, 0.454 mmol) in 4 mL of di­chloro­methane was added to a solution of PhICl_2_ (125 mg, 0.454 mmol) in 3 mL of di­chloro­methane. 2 mL of the mixed solution were divided amongst five small test-tubes and overlayered with various precipitants. The tubes were then stoppered and stored in a refrigerator at 276 K. The measured crystal was obtained using diisopropyl ether as precipitant. Elemental analysis [%]: calc. C 17.91, H 3.26, N 3.48; found C 17.64, H 3.30, N 3.65.

*Tri­bromido­(4-methyl­piperidine)­gold(III)* (**2**)

Polymorph **2a**: Bis(4-methyl­piperidinium) bromide tetra­bromido­aurate(III), {(4-Me-pip)H}_2_·Br·[AuBr_4_] (Döring, 2016 ▸) (26 mg, 0.0327 mmol) was dissolved in 1.5 mL of di­chloro­methane. The solution was divided amongst three small test tubes and overlayered with various precipitants. The tubes were then stoppered and stored in a refrigerator at 276 K. Using diisopropyl ether as precipitant, a mixture of crystals of the starting material (structure to be reported elsewhere) and of **2a** was obtained.

Polymorph **2b**: Bis(4-methyl­piperidine)­gold(I) di­bromido­aurate(I), [(4-Me-pip)_2_Au][AuBr_2_], (90 mg, 0.239 mmol) was dissolved in 2 mL of di­chloro­methane and two drops of elemental bromine were added. The solution was overlayered with diisopropyl ether and stored in a refrigerator at 276 K, whereby crystals of **2b** formed.

## 6. Refinement

Details of the measurements and refinements are given in Table 7 ▸. Structures were refined anisotropically on *F* ^2^. For all compounds, the NH hydrogen atoms were refined freely. Methyl­ene hydrogens were included at calculated positions and refined using a riding model with C—H = 0.99 Å and H—C—H = 109.5°. Methine hydrogens were included similarly, but with C—H = 0.99 Å. Methyl groups were included as idealized rigid groups with C—H 0.98 Å and H—C—H 109.5°, and were allowed to rotate but not to tip (command ‘AFIX 137’). *U* values of the hydrogen atoms were fixed at 1.5 × *U*_eq_ of the parent carbon atoms for methyl groups and 1.2 × *U*_eq_ of the parent carbon atoms for other hydrogens. For compound **2a**, an extinction correction was performed; the extinction parameter (Sheldrick, 2015 ▸) refined to 0.00051 (4).

## Supplementary Material

Crystal structure: contains datablock(s) 1, 2a, 2b, global. DOI: 10.1107/S2056989024002822/pk2705sup1.cif

Structure factors: contains datablock(s) 1. DOI: 10.1107/S2056989024002822/pk27051sup2.hkl

Structure factors: contains datablock(s) 2a. DOI: 10.1107/S2056989024002822/pk27052asup3.hkl

Structure factors: contains datablock(s) 2b. DOI: 10.1107/S2056989024002822/pk27052bsup4.hkl

CCDC references: 2113947, 2113948, 2113946

Additional supporting information:  crystallographic information; 3D view; checkCIF report

## Figures and Tables

**Figure 2:**
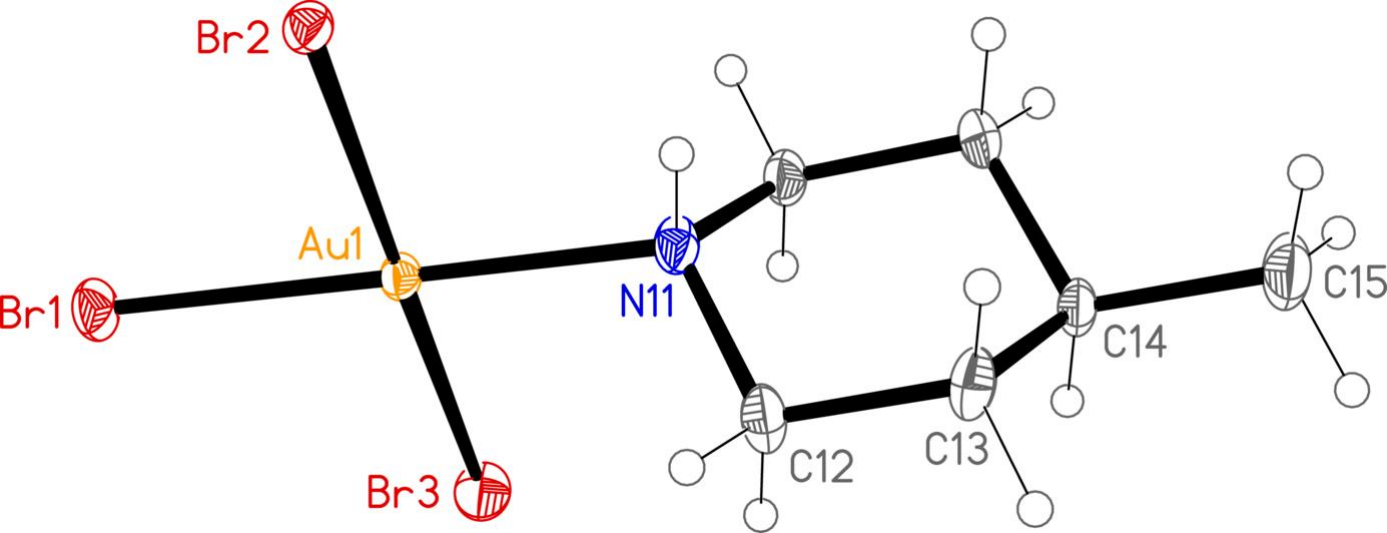
The structure of compound **2a** in the crystal. Ellipsoids correspond to 50% probability levels. Only the asymmetric unit is numbered.

**Figure 3:**
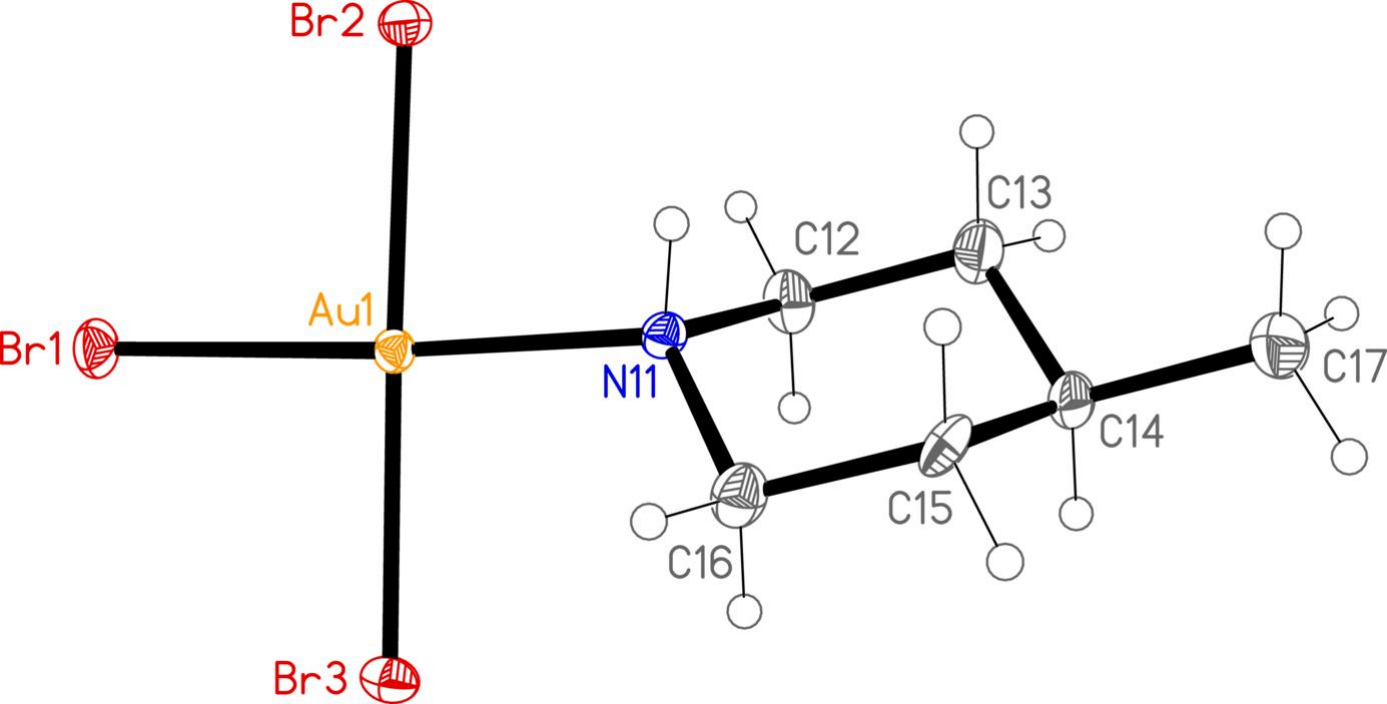
The structure of compound **2b** in the crystal. Ellipsoids correspond to 50% probability levels.

**Table 1:** Selected geometric parameters (Å, °) for **1** [Chem scheme1]

Au1—N11	2.070 (3)	Au1—Cl2	2.2832 (10)
Au1—Cl3	2.2826 (10)	Au1—Cl1	2.3006 (10)
			
N11—Au1—Cl3	93.13 (11)	Cl2—Au1—Cl1	91.18 (4)
N11—Au1—Cl2	85.80 (11)	C16—N11—C12	110.6 (3)
Cl3—Au1—Cl2	178.07 (4)	C16—N11—Au1	117.8 (3)
N11—Au1—Cl1	176.94 (11)	C12—N11—Au1	111.0 (3)
Cl3—Au1—Cl1	89.90 (4)		
			
Cl3—Au1—N11—C16	−30.0 (3)	Au1—N11—C12—C13	169.1 (3)
Cl2—Au1—N11—C16	151.6 (3)	C12—C13—C14—C17	179.8 (4)
Cl3—Au1—N11—C12	98.8 (3)	C17—C14—C15—C16	179.4 (4)
Cl2—Au1—N11—C12	−79.6 (3)	Au1—N11—C16—C15	−172.6 (3)

**Table 2:** Selected geometric parameters (Å, °) for **2a** [Chem scheme1]

Au1—N11	2.096 (5)	Au1—Br3	2.4110 (7)
Au1—Br1	2.4066 (7)	Au1—Br2	2.4273 (6)
			
N11—Au1—Br1	179.50 (15)	Br1—Au1—Br2	91.68 (2)
N11—Au1—Br3	91.78 (15)	Br3—Au1—Br2	179.60 (2)
Br1—Au1—Br3	88.72 (2)	C12^i^—N11—C12	111.2 (5)
N11—Au1—Br2	87.82 (15)	C12^i^—N11—Au1	113.4 (3)
			
Br3—Au1—N11—C12	−64.1 (3)	Au1—N11—C12—C13	−174.4 (3)
Br2—Au1—N11—C12	115.9 (3)	C12—C13—C14—C15	−179.7 (4)

Symmetry code: (i) 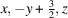 .

**Table 3:** Selected geometric parameters (Å, °) for **2b** [Chem scheme1]

Au1—N11	2.094 (4)	Au1—Br2	2.4244 (5)
Au1—Br1	2.4187 (5)	Au1—Br3	2.4246 (5)
			
N11—Au1—Br1	176.50 (11)	Br2—Au1—Br3	177.736 (17)
N11—Au1—Br2	86.00 (11)	C12—N11—C16	110.9 (4)
Br1—Au1—Br2	90.665 (18)	C12—N11—Au1	111.6 (3)
N11—Au1—Br3	93.62 (11)	C16—N11—Au1	117.8 (3)
Br1—Au1—Br3	89.751 (18)		
			
Br2—Au1—N11—C12	−78.0 (3)	Au1—N11—C12—C13	168.1 (3)
Br3—Au1—N11—C12	99.8 (3)	C12—C13—C14—C17	−179.2 (4)
Br2—Au1—N11—C16	152.0 (3)	C17—C14—C15—C16	178.9 (4)
Br3—Au1—N11—C16	−30.2 (3)	Au1—N11—C16—C15	−171.6 (3)

**Table 4:** Hydrogen-bond geometry (Å, °) for **1** [Chem scheme1]

*D*—H⋯*A*	*D*—H	H⋯*A*	*D*⋯*A*	*D*—H⋯*A*
N11—H01⋯Cl1^i^	0.93 (4)	2.64 (4)	3.535 (4)	163 (4)
C15—H15*B*⋯Cl1^i^	0.99	2.97	3.804 (4)	143
C13—H13*B*⋯Cl2^ii^	0.99	2.82	3.798 (4)	171
C15—H15*A*⋯Cl3^iii^	0.99	2.95	3.610 (4)	125
C15—H15*A*⋯Cl3^iv^	0.99	2.99	3.728 (4)	132
C16—H16*B*⋯Cl3^i^	0.99	2.91	3.656 (5)	133

Symmetry codes: (i) 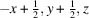 ; (ii) 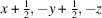 ; (iii) 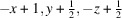 ; (iv) 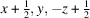 .

**Table 5:** Hydrogen-bond geometry (Å, °) for **2a** [Chem scheme1]

*D*—H⋯*A*	*D*—H	H⋯*A*	*D*⋯*A*	*D*—H⋯*A*
N11—H01⋯Br2	0.89 (6)	2.71 (6)	3.146 (5)	111 (5)
C12—H12*B*⋯Br1^ii^	0.99	2.94	3.786 (5)	145
C12—H12*B*⋯Br2^ii^	0.99	2.99	3.798 (4)	139
C15—H15*A*⋯Br2^iii^	0.97	2.98	3.936 (7)	169
C12—H12*A*⋯Br3	0.99	2.96	3.526 (4)	118
C13—H13*A*⋯Br3^iv^	0.99	3.09	4.002 (4)	154
C15—H15*B*⋯Br3^iv^	0.98	3.05	3.965 (3)	155

Symmetry codes: (ii) 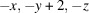 ; (iii) 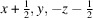 ; (iv) 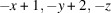 .

**Table 6:** Hydrogen-bond geometry (Å, °) for **2b** [Chem scheme1]

*D*—H⋯*A*	*D*—H	H⋯*A*	*D*⋯*A*	*D*—H⋯*A*
N11—H01⋯Br1^i^	0.97 (4)	2.81 (4)	3.759 (4)	164 (4)
C12—H12*A*⋯Br2	0.99	2.99	3.542 (5)	116
C13—H13*B*⋯Br2^ii^	0.99	2.93	3.903 (5)	169
C16—H16*B*⋯Br3^i^	0.99	2.99	3.750 (5)	135

Symmetry codes: (i) 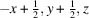 ; (ii) 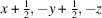 .

**Table 7:** Experimental details

	**1**	**2a**	**2b**
Crystal data			
Chemical formula	[AuCl_3_(C_6_H_13_N)]	[AuBr_3_(C_6_H_13_N)]	[AuBr_3_(C_6_H_13_N)]
*M*_r_	402.49	535.87	535.87
Crystal system, space group	Orthorhombic, *P* *b* *c* *a*	Orthorhombic, *P* *n* *m* *a*	Orthorhombic, *P* *b* *c* *a*
Temperature (K)	100	100	100
*a*, *b*, *c* (Å)	12.5716 (6), 8.3940 (3), 20.3319 (7)	9.9871 (5), 7.1505 (4), 15.7160 (8)	12.6471 (5), 8.7247 (3), 21.0262 (7)
*V* (Å^3^)	2145.53 (14)	1122.32 (10)	2320.07 (15)
*Z*	8	4	8
Radiation type	Mo *K*α	Mo *K*α	Mo *K*α
μ (mm^−1^)	14.40	23.74	22.96
Crystal size (mm)	0.22 × 0.03 × 0.01	0.27 × 0.06 × 0.03	0.14 × 0.04 × 0.03

Data collection			
Diffractometer	Oxford Diffraction Xcalibur, Eos	Oxford Diffraction Xcalibur, Eos	Oxford Diffraction Xcalibur, Eos
Absorption correction	Multi-scan (*CrysAlis PRO*; Rigaku OD, 2015 ▸)	Multi-scan (*CrysAlis PRO*; Rigaku OD, 2015 ▸)	Multi-scan (*CrysAlis PRO*; Rigaku OD, 2015 ▸)
*T*_min_, *T*_max_	0.702, 1.000	0.240, 1.000	0.380, 1.000
No. of measured, independent and observed [*I* > 2σ(*I*)] reflections	53277, 2887, 2134	28605, 1864, 1581	38297, 3371, 2495
*R*_int_	0.080	0.070	0.074
θ values (°)	θ_max_ = 29.1, θ_min_ = 2.6	θ_max_ = 31.1, θ_min_ = 2.4	θ_max_ = 30.0, θ_min_ = 2.5

Refinement			
*R*[*F* ^2^ > 2σ(*F* ^2^)], *wR*(*F* ^2^), *S*	0.024, 0.042, 1.05	0.030, 0.050, 1.11	0.029, 0.043, 1.04
No. of reflections	2887	1864	3371
No. of parameters	105	65	105
H-atom treatment	H atoms treated by a mixture of independent and constrained refinement	H atoms treated by a mixture of independent and constrained refinement	H atoms treated by a mixture of independent and constrained refinement
Δρ_max_, Δρ_min_ (e Å^−3^)	0.86, −0.86	1.56, −1.19	0.99, −0.99

Computer programs: *CrysAlis PRO* (Rigaku OD, 2015 ▸), *SHELXS97* (Sheldrick, 2008 ▸), *SHELXL2019/3* (Sheldrick, 2015 ▸) and *XP*, (Bruker, 1998 ▸).

## supplementary crystallographic information

### Trichlorido(4-methylpiperidine)gold(III) (1) Crystal data

**Table:**

[AuCl_3_(C_6_H_13_N)]	*D*_x_ = 2.492 Mg m^−^^3^
*M**_r_* = 402.49	Mo *K*α radiation, λ = 0.71073 Å
Orthorhombic, *P**b**c**a*	Cell parameters from 7041 reflections
*a* = 12.5716 (6) Å	θ = 2.6–30.3°
*b* = 8.3940 (3) Å	µ = 14.40 mm^−^^1^
*c* = 20.3319 (7) Å	*T* = 100 K
*V* = 2145.53 (14) Å^3^	Needle, yellow
*Z* = 8	0.22 × 0.03 × 0.01 mm
*F*(000) = 1488	

### Trichlorido(4-methylpiperidine)gold(III) (1) Data collection

**Table:**

Oxford Diffraction Xcalibur, Eos diffractometer	2887 independent reflections
Radiation source: Enhance (Mo) X-ray Source	2134 reflections with *I* > 2σ(*I*)
Detector resolution: 16.1419 pixels mm^-1^	*R*_int_ = 0.080
ω scan	θ_max_ = 29.1°, θ_min_ = 2.6°
Absorption correction: multi-scan (CrysAlisPro; Rigaku OD, 2015)	*h* = −17→17
*T*_min_ = 0.702, *T*_max_ = 1.000	*k* = −11→11
53277 measured reflections	*l* = −27→27

### Trichlorido(4-methylpiperidine)gold(III) (1) Refinement

**Table:**

Refinement on *F*^2^	Primary atom site location: structure-invariant direct methods
Least-squares matrix: full	Secondary atom site location: difference Fourier map
*R*[*F*^2^ > 2σ(*F*^2^)] = 0.024	Hydrogen site location: mixed
*wR*(*F*^2^) = 0.042	H atoms treated by a mixture of independent and constrained refinement
*S* = 1.05	*w* = 1/[σ^2^(*F*_o_^2^) + (0.0105*P*)^2^ + 2.0478*P*] where *P* = (*F*_o_^2^ + 2*F*_c_^2^)/3
2887 reflections	(Δ/σ)_max_ = 0.001
105 parameters	Δρ_max_ = 0.86 e Å^−^^3^
0 restraints	Δρ_min_ = −0.86 e Å^−^^3^

### Trichlorido(4-methylpiperidine)gold(III) (1) Fractional atomic coordinates and isotropic or equivalent isotropic displacement parameters (Å^2^)

**Table:**

	*x*	*y*	*z*	*U*_iso_*/*U*_eq_	
Au1	0.24309 (2)	0.30864 (2)	0.12540 (2)	0.01126 (5)	
Cl1	0.06218 (8)	0.27039 (12)	0.11903 (6)	0.0183 (2)	
Cl2	0.23963 (8)	0.47300 (12)	0.03594 (5)	0.0152 (2)	
Cl3	0.25068 (9)	0.13911 (12)	0.21305 (5)	0.0163 (2)	
N11	0.4051 (3)	0.3526 (4)	0.12740 (19)	0.0140 (7)	
H01	0.413 (3)	0.459 (5)	0.116 (2)	0.016 (12)*	
C12	0.4627 (4)	0.2518 (5)	0.0768 (2)	0.0164 (10)	
H12A	0.461894	0.138753	0.090652	0.020*	
H12B	0.426095	0.260030	0.033863	0.020*	
C13	0.5764 (3)	0.3084 (5)	0.0700 (2)	0.0172 (9)	
H13A	0.576749	0.418818	0.052940	0.021*	
H13B	0.613978	0.240127	0.037781	0.021*	
C14	0.6356 (3)	0.3033 (5)	0.1356 (2)	0.0171 (9)	
H14	0.637276	0.190175	0.150987	0.020*	
C15	0.5748 (4)	0.4004 (5)	0.1862 (2)	0.0167 (9)	
H15A	0.610893	0.391913	0.229285	0.020*	
H15B	0.575131	0.513867	0.172913	0.020*	
C16	0.4608 (3)	0.3441 (5)	0.1930 (2)	0.0183 (10)	
H16A	0.459812	0.233002	0.209420	0.022*	
H16B	0.422949	0.411771	0.225335	0.022*	
C17	0.7505 (3)	0.3604 (5)	0.1278 (2)	0.0226 (9)	
H17A	0.750776	0.472073	0.113702	0.034*	
H17B	0.786546	0.295036	0.094620	0.034*	
H17C	0.787735	0.350621	0.169907	0.034*	

### Trichlorido(4-methylpiperidine)gold(III) (1) Atomic displacement parameters (Å^2^)

**Table:**

	*U*^11^	*U*^22^	*U*^33^	*U*^12^	*U*^13^	*U*^23^
Au1	0.01234 (7)	0.00980 (8)	0.01163 (7)	0.00022 (6)	0.00052 (7)	−0.00063 (6)
Cl1	0.0153 (5)	0.0172 (5)	0.0225 (6)	−0.0036 (4)	0.0014 (5)	0.0009 (5)
Cl2	0.0187 (5)	0.0130 (5)	0.0139 (5)	−0.0011 (4)	−0.0021 (4)	0.0019 (4)
Cl3	0.0227 (5)	0.0127 (5)	0.0135 (4)	−0.0006 (4)	0.0019 (5)	0.0015 (4)
N11	0.0121 (16)	0.0110 (17)	0.0189 (18)	0.0001 (13)	−0.0004 (16)	0.0011 (16)
C12	0.014 (2)	0.021 (3)	0.015 (2)	0.0004 (17)	0.0039 (19)	−0.0070 (17)
C13	0.013 (2)	0.023 (3)	0.016 (2)	0.001 (2)	0.0022 (17)	−0.0025 (19)
C14	0.013 (2)	0.020 (2)	0.019 (2)	−0.0010 (18)	−0.0001 (17)	0.0024 (19)
C15	0.018 (2)	0.019 (2)	0.013 (2)	0.0014 (18)	−0.0065 (18)	−0.0034 (17)
C16	0.018 (2)	0.025 (3)	0.012 (2)	−0.0002 (19)	−0.0024 (17)	−0.0042 (18)
C17	0.019 (2)	0.026 (2)	0.022 (2)	−0.0043 (18)	−0.004 (2)	0.007 (2)

### Trichlorido(4-methylpiperidine)gold(III) (1) Geometric parameters (Å, º)

**Table:**

Au1—N11	2.070 (3)	C13—H13B	0.9900
Au1—Cl3	2.2826 (10)	C14—C15	1.518 (6)
Au1—Cl2	2.2832 (10)	C14—C17	1.531 (6)
Au1—Cl1	2.3006 (10)	C14—H14	1.0000
N11—C16	1.509 (5)	C15—C16	1.515 (6)
N11—C12	1.516 (5)	C15—H15A	0.9900
N11—H01	0.93 (4)	C15—H15B	0.9900
C12—C13	1.512 (6)	C16—H16A	0.9900
C12—H12A	0.9900	C16—H16B	0.9900
C12—H12B	0.9900	C17—H17A	0.9800
C13—C14	1.528 (6)	C17—H17B	0.9800
C13—H13A	0.9900	C17—H17C	0.9800
			
N11—Au1—Cl3	93.13 (11)	C15—C14—C13	109.4 (3)
N11—Au1—Cl2	85.80 (11)	C15—C14—C17	112.2 (4)
Cl3—Au1—Cl2	178.07 (4)	C13—C14—C17	111.1 (3)
N11—Au1—Cl1	176.94 (11)	C15—C14—H14	108.0
Cl3—Au1—Cl1	89.90 (4)	C13—C14—H14	108.0
Cl2—Au1—Cl1	91.18 (4)	C17—C14—H14	108.0
C16—N11—C12	110.6 (3)	C16—C15—C14	111.8 (3)
C16—N11—Au1	117.8 (3)	C16—C15—H15A	109.3
C12—N11—Au1	111.0 (3)	C14—C15—H15A	109.3
C16—N11—H01	103 (3)	C16—C15—H15B	109.3
C12—N11—H01	108 (3)	C14—C15—H15B	109.3
Au1—N11—H01	106 (3)	H15A—C15—H15B	107.9
C13—C12—N11	109.8 (3)	N11—C16—C15	110.0 (3)
C13—C12—H12A	109.7	N11—C16—H16A	109.7
N11—C12—H12A	109.7	C15—C16—H16A	109.7
C13—C12—H12B	109.7	N11—C16—H16B	109.7
N11—C12—H12B	109.7	C15—C16—H16B	109.7
H12A—C12—H12B	108.2	H16A—C16—H16B	108.2
C12—C13—C14	111.8 (3)	C14—C17—H17A	109.5
C12—C13—H13A	109.3	C14—C17—H17B	109.5
C14—C13—H13A	109.3	H17A—C17—H17B	109.5
C12—C13—H13B	109.3	C14—C17—H17C	109.5
C14—C13—H13B	109.3	H17A—C17—H17C	109.5
H13A—C13—H13B	107.9	H17B—C17—H17C	109.5
			
Cl3—Au1—N11—C16	−30.0 (3)	C12—C13—C14—C15	−55.8 (5)
Cl2—Au1—N11—C16	151.6 (3)	C12—C13—C14—C17	179.8 (4)
Cl3—Au1—N11—C12	98.8 (3)	C13—C14—C15—C16	55.7 (5)
Cl2—Au1—N11—C12	−79.6 (3)	C17—C14—C15—C16	179.4 (4)
C16—N11—C12—C13	−58.4 (4)	C12—N11—C16—C15	58.4 (4)
Au1—N11—C12—C13	169.1 (3)	Au1—N11—C16—C15	−172.6 (3)
N11—C12—C13—C14	57.4 (5)	C14—C15—C16—N11	−57.7 (5)

### Trichlorido(4-methylpiperidine)gold(III) (1) Hydrogen-bond geometry (Å, º)

**Table:**

*D*—H···*A*	*D*—H	H···*A*	*D*···*A*	*D*—H···*A*
N11—H01···Cl1^i^	0.93 (4)	2.64 (4)	3.535 (4)	163 (4)
C15—H15*B*···Cl1^i^	0.99	2.97	3.804 (4)	143
C13—H13*B*···Cl2^ii^	0.99	2.82	3.798 (4)	171
C15—H15*A*···Cl3^iii^	0.99	2.95	3.610 (4)	125
C15—H15*A*···Cl3^iv^	0.99	2.99	3.728 (4)	132
C16—H16*B*···Cl3^i^	0.99	2.91	3.656 (5)	133

Symmetry codes: (i) −*x*+1/2, *y*+1/2, *z*; (ii) *x*+1/2, −*y*+1/2, −*z*; (iii) −*x*+1, *y*+1/2, −*z*+1/2; (iv) *x*+1/2, *y*, −*z*+1/2.

### Tribromido(4-methylpiperidine)gold(III) (2a) Crystal data

**Table:**

[AuBr_3_(C_6_H_13_N)]	*D*_x_ = 3.171 Mg m^−^^3^
*M**_r_* = 535.87	Mo *K*α radiation, λ = 0.71073 Å
Orthorhombic, *P**n**m**a*	Cell parameters from 4663 reflections
*a* = 9.9871 (5) Å	θ = 3.1–30.3°
*b* = 7.1505 (4) Å	µ = 23.74 mm^−^^1^
*c* = 15.7160 (8) Å	*T* = 100 K
*V* = 1122.32 (10) Å^3^	Needle, orange
*Z* = 4	0.27 × 0.06 × 0.03 mm
*F*(000) = 960	

### Tribromido(4-methylpiperidine)gold(III) (2a) Data collection

**Table:**

Oxford Diffraction Xcalibur, Eos diffractometer	1864 independent reflections
Radiation source: fine-focus sealed X-ray tube	1581 reflections with *I* > 2σ(*I*)
Detector resolution: 16.1419 pixels mm^-1^	*R*_int_ = 0.070
ω scan	θ_max_ = 31.1°, θ_min_ = 2.4°
Absorption correction: multi-scan (CrysAlisPro; Rigaku OD, 2015)	*h* = −14→14
*T*_min_ = 0.240, *T*_max_ = 1.000	*k* = −10→10
28605 measured reflections	*l* = −22→22

### Tribromido(4-methylpiperidine)gold(III) (2a) Refinement

**Table:**

Refinement on *F*^2^	Secondary atom site location: difference Fourier map
Least-squares matrix: full	Hydrogen site location: mixed
*R*[*F*^2^ > 2σ(*F*^2^)] = 0.030	H atoms treated by a mixture of independent and constrained refinement
*wR*(*F*^2^) = 0.050	*w* = 1/[σ^2^(*F*_o_^2^) + (0.0121*P*)^2^ + 3.4353*P*] where *P* = (*F*_o_^2^ + 2*F*_c_^2^)/3
*S* = 1.11	(Δ/σ)_max_ < 0.001
1864 reflections	Δρ_max_ = 1.56 e Å^−^^3^
65 parameters	Δρ_min_ = −1.18 e Å^−^^3^
0 restraints	Extinction correction: *SHELXL2019/3* (Sheldrick, 2015), Fc^*^=kFc[1+0.001xFc^2^λ^3^/sin(2θ)]^-1/4^
Primary atom site location: structure-invariant direct methods	Extinction coefficient: 0.00051 (4)

### Tribromido(4-methylpiperidine)gold(III) (2a) Fractional atomic coordinates and isotropic or equivalent isotropic displacement parameters (Å^2^)

**Table:**

	*x*	*y*	*z*	*U*_iso_*/*U*_eq_	
Au1	0.09315 (2)	0.750000	0.04663 (2)	0.01176 (7)	
Br1	−0.07000 (6)	0.750000	0.15933 (4)	0.01882 (14)	
Br2	−0.08081 (6)	0.750000	−0.06122 (4)	0.01351 (13)	
Br3	0.26712 (6)	0.750000	0.15298 (4)	0.02039 (15)	
N11	0.2339 (5)	0.750000	−0.0523 (3)	0.0161 (11)	
H01	0.184 (7)	0.750000	−0.099 (4)	0.013 (17)*	
C12	0.3183 (4)	0.9225 (6)	−0.0547 (3)	0.0182 (9)	
H12A	0.373767	0.928684	−0.002574	0.022*	
H12B	0.259617	1.034089	−0.055751	0.022*	
C13	0.4087 (4)	0.9241 (6)	−0.1324 (3)	0.0193 (9)	
H13A	0.465879	1.037237	−0.131098	0.023*	
H13B	0.353144	0.929562	−0.184542	0.023*	
C14	0.4975 (6)	0.750000	−0.1354 (4)	0.0152 (12)	
H14	0.554971	0.750002	−0.083321	0.018*	
C15	0.5896 (7)	0.750000	−0.2129 (4)	0.0253 (16)	
H15A	0.534829	0.750000	−0.264018	0.038*	
H15B	0.647617	0.860443	−0.211145	0.038*	

### Tribromido(4-methylpiperidine)gold(III) (2a) Atomic displacement parameters (Å^2^)

**Table:**

	*U*^11^	*U*^22^	*U*^33^	*U*^12^	*U*^13^	*U*^23^
Au1	0.01034 (11)	0.01467 (12)	0.01027 (12)	0.000	0.00112 (9)	0.000
Br1	0.0180 (3)	0.0253 (3)	0.0132 (3)	0.000	0.0057 (2)	0.000
Br2	0.0113 (3)	0.0163 (3)	0.0129 (3)	0.000	−0.0005 (2)	0.000
Br3	0.0172 (3)	0.0304 (4)	0.0135 (3)	0.000	−0.0042 (2)	0.000
N11	0.011 (2)	0.027 (3)	0.010 (3)	0.000	0.000 (2)	0.000
C12	0.018 (2)	0.016 (2)	0.021 (2)	0.0031 (17)	0.0090 (18)	0.0020 (19)
C13	0.016 (2)	0.022 (2)	0.020 (2)	−0.0009 (19)	0.0067 (19)	0.005 (2)
C14	0.013 (3)	0.016 (3)	0.017 (3)	0.000	0.007 (2)	0.000
C15	0.017 (3)	0.033 (4)	0.026 (4)	0.000	0.009 (3)	0.000

### Tribromido(4-methylpiperidine)gold(III) (2a) Geometric parameters (Å, º)

**Table:**

Au1—N11	2.096 (5)	C12—H12B	0.9900
Au1—Br1	2.4066 (7)	C13—C14	1.529 (5)
Au1—Br3	2.4110 (7)	C13—H13A	0.9900
Au1—Br2	2.4273 (6)	C13—H13B	0.9900
N11—C12^i^	1.494 (5)	C14—C15	1.526 (8)
N11—C12	1.494 (5)	C14—H14	1.0000
N11—H01	0.89 (6)	C15—H15A	0.9713
C12—C13	1.520 (6)	C15—H15B	0.9800
C12—H12A	0.9900	C15—H15B^i^	0.9800
			
N11—Au1—Br1	179.50 (15)	C12—C13—C14	111.3 (4)
N11—Au1—Br3	91.78 (15)	C12—C13—H13A	109.4
Br1—Au1—Br3	88.72 (2)	C14—C13—H13A	109.4
N11—Au1—Br2	87.82 (15)	C12—C13—H13B	109.4
Br1—Au1—Br2	91.68 (2)	C14—C13—H13B	109.4
Br3—Au1—Br2	179.60 (2)	H13A—C13—H13B	108.0
C12^i^—N11—C12	111.2 (5)	C15—C14—C13^i^	111.9 (3)
C12^i^—N11—Au1	113.4 (3)	C15—C14—C13	111.9 (3)
C12—N11—Au1	113.4 (3)	C13^i^—C14—C13	109.0 (5)
C12^i^—N11—H01	107 (2)	C15—C14—H14	107.9
C12—N11—H01	107 (2)	C13^i^—C14—H14	107.9
Au1—N11—H01	104 (4)	C13—C14—H14	107.9
N11—C12—C13	111.2 (4)	C14—C15—H15A	108.7
N11—C12—H12A	109.4	C14—C15—H15B	109.5
C13—C12—H12A	109.4	H15A—C15—H15B	110.9
N11—C12—H12B	109.4	C14—C15—H15B^i^	109.48 (19)
C13—C12—H12B	109.4	H15A—C15—H15B^i^	110.9
H12A—C12—H12B	108.0	H15B—C15—H15B^i^	107.4
			
Br3—Au1—N11—C12^i^	64.1 (3)	Au1—N11—C12—C13	−174.4 (3)
Br2—Au1—N11—C12^i^	−115.9 (3)	N11—C12—C13—C14	−56.8 (5)
Br3—Au1—N11—C12	−64.1 (3)	C12—C13—C14—C15	−179.7 (4)
Br2—Au1—N11—C12	115.9 (3)	C12—C13—C14—C13^i^	55.9 (6)
C12^i^—N11—C12—C13	56.4 (6)		

Symmetry code: (i) *x*, −*y*+3/2, *z*.

### Tribromido(4-methylpiperidine)gold(III) (2a) Hydrogen-bond geometry (Å, º)

**Table:**

*D*—H···*A*	*D*—H	H···*A*	*D*···*A*	*D*—H···*A*
N11—H01···Br2	0.89 (6)	2.71 (6)	3.146 (5)	111 (5)
C12—H12*B*···Br1^ii^	0.99	2.94	3.786 (5)	145
C12—H12*B*···Br2^ii^	0.99	2.99	3.798 (4)	139
C15—H15*A*···Br2^iii^	0.97	2.98	3.936 (7)	169
C12—H12*A*···Br3	0.99	2.96	3.526 (4)	118
C13—H13*A*···Br3^iv^	0.99	3.09	4.002 (4)	154
C15—H15*B*···Br3^iv^	0.98	3.05	3.965 (3)	155

Symmetry codes: (ii) −*x*, −*y*+2, −*z*; (iii) *x*+1/2, *y*, −*z*−1/2; (iv) −*x*+1, −*y*+2, −*z*.

### Tribromido(4-methylpiperidine)gold(III) (2b) Crystal data

**Table:**

[AuBr_3_(C_6_H_13_N)]	*D*_x_ = 3.068 Mg m^−^^3^
*M**_r_* = 535.87	Mo *K*α radiation, λ = 0.71073 Å
Orthorhombic, *P**b**c**a*	Cell parameters from 5682 reflections
*a* = 12.6471 (5) Å	θ = 3.2–29.7°
*b* = 8.7247 (3) Å	µ = 22.96 mm^−^^1^
*c* = 21.0262 (7) Å	*T* = 100 K
*V* = 2320.07 (15) Å^3^	Needle, red
*Z* = 8	0.14 × 0.04 × 0.03 mm
*F*(000) = 1920	

### Tribromido(4-methylpiperidine)gold(III) (2b) Data collection

**Table:**

Oxford Diffraction Xcalibur, Eos diffractometer	3371 independent reflections
Radiation source: Enhance (Mo) X-ray Source	2495 reflections with *I* > 2σ(*I*)
Detector resolution: 16.1419 pixels mm^-1^	*R*_int_ = 0.074
ω scan	θ_max_ = 30.0°, θ_min_ = 2.5°
Absorption correction: multi-scan (CrysAlisPro; Rigaku OD, 2015)	*h* = −17→17
*T*_min_ = 0.380, *T*_max_ = 1.000	*k* = −11→12
38297 measured reflections	*l* = −29→29

### Tribromido(4-methylpiperidine)gold(III) (2b) Refinement

**Table:**

Refinement on *F*^2^	Primary atom site location: structure-invariant direct methods
Least-squares matrix: full	Secondary atom site location: difference Fourier map
*R*[*F*^2^ > 2σ(*F*^2^)] = 0.029	Hydrogen site location: mixed
*wR*(*F*^2^) = 0.043	H atoms treated by a mixture of independent and constrained refinement
*S* = 1.03	*w* = 1/[σ^2^(*F*_o_^2^) + (0.0081*P*)^2^ + 2.1449*P*] where *P* = (*F*_o_^2^ + 2*F*_c_^2^)/3
3371 reflections	(Δ/σ)_max_ = 0.001
105 parameters	Δρ_max_ = 0.99 e Å^−^^3^
0 restraints	Δρ_min_ = −0.99 e Å^−^^3^

### Tribromido(4-methylpiperidine)gold(III) (2b) Fractional atomic coordinates and isotropic or equivalent isotropic displacement parameters (Å^2^)

**Table:**

	*x*	*y*	*z*	*U*_iso_*/*U*_eq_	
Au1	0.24373 (2)	0.30618 (2)	0.12636 (2)	0.01070 (5)	
Br1	0.05410 (4)	0.27328 (5)	0.12037 (2)	0.01833 (11)	
Br2	0.24027 (4)	0.47282 (5)	0.03411 (2)	0.01409 (10)	
Br3	0.24951 (4)	0.13133 (5)	0.21592 (2)	0.01560 (10)	
N11	0.4069 (3)	0.3465 (4)	0.12801 (19)	0.0115 (8)	
H01	0.416 (4)	0.454 (5)	0.116 (2)	0.019 (14)*	
C12	0.4638 (4)	0.2527 (5)	0.0784 (2)	0.0169 (11)	
H12A	0.426683	0.261747	0.037140	0.020*	
H12B	0.463810	0.143443	0.091066	0.020*	
C13	0.5769 (4)	0.3088 (6)	0.0714 (2)	0.0201 (11)	
H13A	0.576259	0.415630	0.055540	0.024*	
H13B	0.613889	0.244725	0.039599	0.024*	
C14	0.6373 (4)	0.3028 (5)	0.1341 (2)	0.0162 (10)	
H14	0.640778	0.193362	0.148091	0.019*	
C15	0.5762 (4)	0.3927 (5)	0.1845 (2)	0.0154 (11)	
H15A	0.612751	0.382498	0.225900	0.018*	
H15B	0.575849	0.502588	0.172813	0.018*	
C16	0.4628 (4)	0.3372 (5)	0.1914 (2)	0.0180 (11)	
H16A	0.462434	0.229940	0.206773	0.022*	
H16B	0.425261	0.401276	0.222985	0.022*	
C17	0.7504 (4)	0.3616 (6)	0.1266 (2)	0.0253 (11)	
H17A	0.748980	0.471144	0.116451	0.038*	
H17B	0.785491	0.305515	0.092217	0.038*	
H17C	0.789141	0.345693	0.166448	0.038*	

### Tribromido(4-methylpiperidine)gold(III) (2b) Atomic displacement parameters (Å^2^)

**Table:**

	*U*^11^	*U*^22^	*U*^33^	*U*^12^	*U*^13^	*U*^23^
Au1	0.01025 (9)	0.00994 (8)	0.01190 (8)	0.00053 (7)	0.00084 (8)	−0.00021 (7)
Br1	0.0108 (2)	0.0184 (2)	0.0259 (3)	−0.00129 (18)	0.0015 (2)	0.0018 (2)
Br2	0.0151 (2)	0.0137 (2)	0.0135 (2)	−0.0008 (2)	−0.0013 (2)	0.00201 (17)
Br3	0.0207 (3)	0.0128 (2)	0.0133 (2)	0.0001 (2)	0.0017 (2)	0.00118 (17)
N11	0.011 (2)	0.0125 (19)	0.011 (2)	−0.0006 (15)	−0.0018 (17)	0.0001 (16)
C12	0.016 (3)	0.021 (3)	0.013 (2)	0.001 (2)	0.003 (2)	−0.0075 (19)
C13	0.015 (3)	0.025 (3)	0.021 (3)	0.002 (2)	0.000 (2)	−0.001 (2)
C14	0.011 (2)	0.018 (2)	0.020 (3)	−0.002 (2)	−0.002 (2)	0.002 (2)
C15	0.013 (3)	0.016 (2)	0.017 (3)	0.003 (2)	−0.008 (2)	−0.002 (2)
C16	0.016 (3)	0.021 (3)	0.017 (3)	0.001 (2)	−0.002 (2)	−0.003 (2)
C17	0.018 (3)	0.034 (3)	0.024 (3)	−0.006 (2)	−0.003 (3)	0.007 (2)

### Tribromido(4-methylpiperidine)gold(III) (2b) Geometric parameters (Å, º)

**Table:**

Au1—N11	2.094 (4)	C13—H13B	0.9900
Au1—Br1	2.4187 (5)	C14—C15	1.527 (6)
Au1—Br2	2.4244 (5)	C14—C17	1.527 (6)
Au1—Br3	2.4246 (5)	C14—H14	1.0000
N11—C12	1.508 (6)	C15—C16	1.520 (7)
N11—C16	1.511 (6)	C15—H15A	0.9900
N11—H01	0.97 (4)	C15—H15B	0.9900
C12—C13	1.518 (7)	C16—H16A	0.9900
C12—H12A	0.9900	C16—H16B	0.9900
C12—H12B	0.9900	C17—H17A	0.9800
C13—C14	1.524 (7)	C17—H17B	0.9800
C13—H13A	0.9900	C17—H17C	0.9800
			
N11—Au1—Br1	176.50 (11)	C13—C14—C15	109.2 (4)
N11—Au1—Br2	86.00 (11)	C13—C14—C17	111.6 (4)
Br1—Au1—Br2	90.665 (18)	C15—C14—C17	111.9 (4)
N11—Au1—Br3	93.62 (11)	C13—C14—H14	108.0
Br1—Au1—Br3	89.751 (18)	C15—C14—H14	108.0
Br2—Au1—Br3	177.736 (17)	C17—C14—H14	108.0
C12—N11—C16	110.9 (4)	C16—C15—C14	112.3 (4)
C12—N11—Au1	111.6 (3)	C16—C15—H15A	109.1
C16—N11—Au1	117.8 (3)	C14—C15—H15A	109.1
C12—N11—H01	107 (3)	C16—C15—H15B	109.1
C16—N11—H01	102 (3)	C14—C15—H15B	109.1
Au1—N11—H01	106 (3)	H15A—C15—H15B	107.9
N11—C12—C13	110.0 (4)	N11—C16—C15	109.9 (4)
N11—C12—H12A	109.7	N11—C16—H16A	109.7
C13—C12—H12A	109.7	C15—C16—H16A	109.7
N11—C12—H12B	109.7	N11—C16—H16B	109.7
C13—C12—H12B	109.7	C15—C16—H16B	109.7
H12A—C12—H12B	108.2	H16A—C16—H16B	108.2
C12—C13—C14	112.2 (4)	C14—C17—H17A	109.5
C12—C13—H13A	109.2	C14—C17—H17B	109.5
C14—C13—H13A	109.2	H17A—C17—H17B	109.5
C12—C13—H13B	109.2	C14—C17—H17C	109.5
C14—C13—H13B	109.2	H17A—C17—H17C	109.5
H13A—C13—H13B	107.9	H17B—C17—H17C	109.5
			
Br2—Au1—N11—C12	−78.0 (3)	C12—C13—C14—C15	−55.0 (5)
Br3—Au1—N11—C12	99.8 (3)	C12—C13—C14—C17	−179.2 (4)
Br2—Au1—N11—C16	152.0 (3)	C13—C14—C15—C16	54.8 (5)
Br3—Au1—N11—C16	−30.2 (3)	C17—C14—C15—C16	178.9 (4)
C16—N11—C12—C13	−58.4 (5)	C12—N11—C16—C15	58.0 (5)
Au1—N11—C12—C13	168.1 (3)	Au1—N11—C16—C15	−171.6 (3)
N11—C12—C13—C14	57.4 (5)	C14—C15—C16—N11	−56.8 (5)

### Tribromido(4-methylpiperidine)gold(III) (2b) Hydrogen-bond geometry (Å, º)

**Table:**

*D*—H···*A*	*D*—H	H···*A*	*D*···*A*	*D*—H···*A*
N11—H01···Br1^i^	0.97 (4)	2.81 (4)	3.759 (4)	164 (4)
C12—H12*A*···Br2	0.99	2.99	3.542 (5)	116
C13—H13*B*···Br2^ii^	0.99	2.93	3.903 (5)	169
C16—H16*B*···Br3^i^	0.99	2.99	3.750 (5)	135

Symmetry codes: (i) −*x*+1/2, *y*+1/2, *z*; (ii) *x*+1/2, −*y*+1/2, −*z*.
